# Human Monkeypox in Sierra Leone after 44-Year Absence of Reported Cases

**DOI:** 10.3201/eid2505.180832

**Published:** 2019-05

**Authors:** Mary G. Reynolds, Nadia Wauquier, Yu Li, Panayampalli Subbian Satheshkumar, Lansana D. Kanneh, Benjamin Monroe, Jacob Maikere, Gbessay Saffa, Jean-Paul Gonzalez, Joseph Fair, Darin S. Carroll, Amara Jambai, Foday Dafae, Sheik Humarr Khan, Lina M. Moses

**Affiliations:** Centers for Disease Control and Prevention, Atlanta, Georgia, USA (M.G. Reynolds, Y. Li, P.S. Satheshkumar, B. Monroe, D.S. Carroll);; MRI Global–Global Health Surveillance and Diagnostics, Gaithersburg, Maryland, USA (N. Wauquier);; Kenema Government Hospital, Kenema, Sierra Leone (L.D. Kanneh, S.H. Khan);; Médecins Sans Frontières, Brussels, Belgium (J. Maikere);; Ministry of Health and Sanitation, Bo, Sierra Leone (G. Saffa);; Center of Excellence for Emerging Zoonotic and Animal Diseases, Manhattan, Kansas, USA (J.-P. Gonzalez);; Texas A&M University Agrilife Research, College Station, Texas, USA (J. Fair);; Ministry of Health and Sanitation, Freetown, Sierra Leone (A. Jambai, F. Dafae, S.H. Khan);; Tulane University, New Orleans, Louisiana, USA (L.M. Moses)

**Keywords:** monkeypox, case report, Sierra Leone, orthopoxvirus, epidemiology, viruses

## Abstract

We note the reemergence of human monkeypox in Sierra Leone following a 44-year absence of reported disease. The persons affected were an 11-month-old boy and, several years later, a 35-year-old man. The reappearance of monkeypox in this country suggests a need for renewed vigilance and awareness of the disease and its manifestations.

Monkeypox, a tropical zoonosis with an estimated death rate of 15% in children, is a resurgent presence in several countries in West and Central Africa (*1*,*2*). Before 2000, only 21 cases of monkeypox had been reported from these regions, including a single case in Sierra Leone in 1970 (*3*). The disease had not been observed in Sierra Leone since then, although a 2007 survey for orthopoxvirus antibodies among populations near Kenema, Sierra Leone, generated evidence to suggest ongoing circulation of orthopoxviruses in the area (*4*).

On March 18, 2014, a resident of Kpetema town in Sierra Leone brought her 11-month-old son to the community health post in nearby Mano village. There, he was evaluated for fever and released. The child failed to improve, and the next day his mother again sought medical care, this time from the community health center (CHC) in Koribondo. At this time, the child remained febrile and was exhibiting a nascent-stage rash. At the Koribondo CHC the child was given a presumptive diagnosis of early stage chickenpox and sent home.

Within 2 days of the child’s evaluation at the Koribondo CHC, pustular, umbilicated lesions had spread to cover his body, including his face, mouth, oral mucosa, trunk, back, palms, and genital area. In addition, the child was experiencing sweats, chills, vomiting, loss of appetite, cough, and pruritus. On March 21, the child was taken to the Médicins sans Frontières Gondama Referral Center and was admitted with fever (body temperature 38.9**°**C) and disseminated vesiculopustular rash. Notably, lymphadenopathy was absent. On hospital day 4 (day 8 after fever onset), physicians from Médicins sans Frontières working in a pediatric ward at the facility alerted national authorities that they suspected the child might have monkeypox. After consultation with the Directorate of Disease Prevention and Control of the Sierra Leone Ministry of Health and Sanitation, Kenema scientists contacted the Centers for Disease Control and Prevention (CDC) in Atlanta, Georgia, USA.

Diagnostic specimens were collected from the patient on April 1. At that time his rash was desquamating. Lesion crusts and serum samples were obtained, and specimens were shipped to CDC on April 4. On April 8, real-time PCR testing for nonvariola *Orthopoxvirus* and for West African variant monkeypox virus (*5*) yielded weak positive findings from both the lesion and serum specimens. The patient’s serum sample also tested positive for the presence of *Orthopoxvirus* IgG and IgM (*6*).

No viable virus was detected in the specimens. Further genetic characterization of the virus was unsuccessful owing to the limited quality and quantity of specimens.

Upon confirmation of monkeypox etiology, investigators from the Bo District Surveillance Office and the Lassa Fever Outreach Program traveled to the child’s town (Kpetema; Figure) to interview his parents. By that time, the child had recovered. The child’s mother denied that he had had any contact with persons exhibiting a monkeypox-like illness in the 2 weeks before onset of his illness. The mother also denied that the child had had any history of contact with animals. However, both the mother and father of the boy stated that they regularly prepare and consume meat from wild animals. The mother and father also confirmed that small rodents were sometimes present in the family house.

**Figure:**
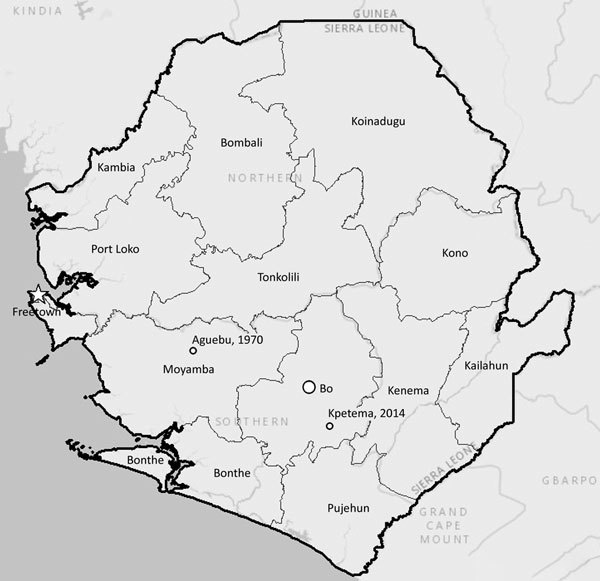
Locations of monkeypox cases in Sierra from 2014 (Kpetema) and 1970 (Aguebu). Map credits: Esri, HERE, Delorme, MapmyIndia, © OpenStreetMap contributors, and the GIS user community.

On March 25, 2017, a 35-year-old male farmer in Pujehun district came to Pujehun Government Hospital in Sierra Leone with fever, generalized body pain, enlarged cervical lymph nodes, dysphagia, malaise, and macular rash with cropping of papules and macules. Various samples were taken and shipped out of the country for laboratory investigations. Monkeypox was confirmed, and community sensitization was instituted. 

The detection of monkeypox virus infection in an 11-month-old child and a 35-year-old man signals the possible reemergence of this disease in Sierra Leone (*3*), from which it had not been reported for 44 years. The reappearance of monkeypox in Sierra Leone suggests a need for renewed vigilance and heightened awareness of the disease and its manifestations. In the early stages of illness, monkeypox can be mistaken for chickenpox, as happened in the case of the child. The confusion led to a delay in diagnosing and reporting the Kpetema case.

Multiple factors are probably driving the resurgence of monkeypox in West and Central Africa. Increasing population susceptibility is likely to be one (*7*). Smallpox vaccine has been shown to confer protection against monkeypox virus infection (*8*). However, routine vaccination ceased worldwide after the declaration by the World Health Organization in 1980 that smallpox had been eradicated. The lack of smallpox vaccination has led to the steady accumulation of poxvirus-susceptible human hosts in the areas of West and Central Africa where humans routinely encounter sylvatic animals, some of which may harbor monkeypox virus. Disease control efforts could be further aided by dedicated surveillance for monkeypox virus among target animal species (*9*).
